# Jasmonate-Mediated Induced Volatiles in the American Cranberry, *Vaccinium macrocarpon*: From Gene Expression to Organismal Interactions

**DOI:** 10.3389/fpls.2013.00115

**Published:** 2013-04-29

**Authors:** Cesar R. Rodriguez-Saona, James Polashock, Edi A. Malo

**Affiliations:** ^1^Department of Entomology, Philip E. Marucci Center for Blueberry and Cranberry ResearchChatsworth, NJ, USA; ^2^Genetic Improvement of Fruits and Vegetables Lab, Philip E. Marucci Center for Blueberry and Cranberry Research, United States Department of Agriculture-Agricultural Research ServiceChatsworth, NJ, USA; ^3^Grupo de Ecología de Artrópodos y Manejo de plagas, El Colegio de la Frontera SurTapachula, Chiapas, México

**Keywords:** methyl jasmonate, jasmonic acid, herbivore-induced plant volatiles, *Sparganothis sulfureana*, electroantennograms, multi-trophic interactions

## Abstract

Jasmonates, i.e., jasmonic acid (JA) and methyl jasmonate (MeJA), are signaling hormones that regulate a large number of defense responses in plants which in turn affect the plants’ interactions with herbivores and their natural enemies. Here, we investigated the effect of jasmonates on the emission of volatiles in the American cranberry, *Vaccinium macrocarpon*, at different levels of biological organization from gene expression to organismal interactions. At the molecular level, four genes (*BCS*, *LLS*, *NER1*, and *TPS21*) responded significantly to gypsy moth larval feeding, MeJA, and mechanical wounding, but to different degrees. The most dramatic changes in expression of *BCS* and *TPS21* (genes in the sesquiterpenoid pathway) were when treated with MeJA. Gypsy moth-damaged and MeJA-treated plants also had significantly elevated expression of *LLS* and *NER1* (genes in the monoterpene and homoterpene biosynthesis pathways, respectively). At the biochemical level, MeJA induced a complex blend of monoterpene and sesquiterpene compounds that differed from gypsy moth and mechanical damage, and followed a diurnal pattern of emission. At the organismal level, numbers of *Sparganothis sulfureana* moths were lower while numbers of parasitic wasps were higher on sticky traps near MeJA-treated cranberry plants than those near untreated plants. Out of 11 leaf volatiles tested, (*Z*)-3-hexenyl acetate, linalool, and linalool oxide elicited strong antennal (EAG) responses from *S. sulfureana*, whereas sesquiterpenes elicited weak EAG responses. In addition, mortality of *S. sulfureana* larvae increased by about 43% in JA treated cranberry plants as compared with untreated plants, indicating a relationship among adult preference, antennal sensitivity to plant odors, and offspring performance. This study highlights the role of the jasmonate-dependent defensive pathway in the emissions of herbivore-induced volatiles in cranberries and its importance in multi-trophic level interactions.

## Introduction

Plants can sometimes change their phenotype after herbivore feeding damage by becoming more protected from future enemy attacks (Karban and Baldwin, 1997; Walling, 2000; Heil, 2010). These induced phenotypic changes can reduce the performance of herbivores or alter their behavior, i.e., reduce the herbivore’s feeding or oviposition on plants (Agrawal, 1998; Denno et al., 2000; De Moraes et al., 2001; Kessler and Baldwin, 2001; Van Zandt and Agrawal, 2004; Viswanathan et al., 2005), and can thus be classified as direct defenses if they provide a fitness benefit to plants (Agrawal, 1998, 2000). They can also indirectly defend plants by changing the behavior of the herbivores’ natural enemies (Price et al., 1980). One such indirect defense is the emission of volatiles – so-called herbivore-induced plant volatiles (HIPVs) – that attract predators and parasitoids of insect herbivores to the attacked plant (Turlings et al., 1990; Vet and Dicke, 1992; Dicke and Vet, 1999). Manipulation of these defensive traits in plants can serve as a natural pest control tactic in agriculture (Thaler, 1999a; Rodriguez-Saona et al., 2012).

An important signaling pathway involved in the induction of plant defenses, including HIPV emissions, is the octadecanoid pathway (Karban and Baldwin, 1997; Arimura et al., 2005). In many plants, activation of the octadecanoid pathway by herbivore feeding may lead to increased production of the plant growth regulator, jasmonic acid (JA) (Farmer et al., 1992; Staswick and Lehman, 1999), which in turn can elicit multiple direct and indirect defenses in plants (Karban and Baldwin, 1997). For example, increases in HIPV emissions similar to those induced by insect feeding have been reported in response to exogenous applications of jasmonates, i.e., JA or its volatile methyl ester methyl jasmonate (MeJA), in many plant species including gerbera, *Gerbera jamesonii* Bolus (Gols et al., 1999), cotton, *Gossypium hirsutum* L. (Rodriguez-Saona et al., 2001), Manchurian ash, *Fraxinus mandshurica* Rupr. (Rodriguez-Saona et al., 2006), and sacred datura, *Datura wrightii* Regel (Hare, 2007).

Activation of the octadecanoid pathway by jasmonate treatment is known to affect the preference and performance of herbivores on plants. Thaler et al. (2001), for example, found fewer caterpillars, flea beetles, aphids, and thrips on tomato plants that were sprayed with JA. *Manduca quinquemaculata* Haworth oviposition was reduced when *Nicotiana attenuata* Torr ex. S. Watts plants were treated with MeJA (Kessler and Baldwin, 2001). Similarly, Bruinsma et al. (2007) showed that two species of cabbage white butterflies, *Pieris rapae* L. and *P. brassicae* L., laid fewer eggs on JA-treated Brussels sprouts, *Brassica oleracea* L., plants compared to untreated plants. *Pieris rapae* also preferred untreated leaves over JA-treated leaves of black mustard plants, *Brassica nigra* L., for oviposition (Bruinsma et al., 2008). Jasmonate-induced changes can also affect members of higher trophic levels. For example, JA treatment increased parasitism of caterpillars in tomato fields (Thaler, 1999b), but negatively affected predatory hoverflies due to a decrease in prey (aphid) abundance (Thaler, 2002). JA or MeJA also induced the emission of plant volatiles that attracted predatory mites (Dicke et al., 1999; Gols et al., 2003).

Studies that integrate multiple research approaches, such as gene expression, metabolite induction, and ecological interactions, are needed for a better understanding of plant interactions with arthropods across different levels of biological organization (Zheng and Dicke, 2008). Previously, we showed that gypsy moth, *Lymantria dispar* L. (Lep., Lymantriidae), larval feeding, and MeJA induce emissions of several monoterpenes and sesquiterpenes in a perennial ericaceous crop, the American cranberry (*Vaccinium macrocarpon* Ait.) (Rodriguez-Saona et al., 2011). In the present study, we linked volatile induction with transcriptional induction and organismal level interactions by investigating the effects of jasmonates on the up-regulation of several key terpene biosynthesis genes, induced volatile emissions, and the preference-performance of a polyphagous herbivore, *Sparganothis sulfureana* Clemens (Sparganothis fruitworm; Lep., Tortricidae), in cranberries. *Sparganothis sulfureana* is an important pest of cranberries in the United States (USA); in spring, the overwintered first instar larvae feed on foliage, adults emerge in late spring to mid-summer, and in June to early July second generation larvae feed on foliage and burrow into developing fruit (Beckwith, 1938; Averill and Sylvia, 1998). Specifically, we conducted studies to: (1) determine the effects of MeJA, gypsy moth larval feeding, and mechanical wounding on expression of eight genes involved in terpenoid biosynthesis; (2) compare the volatile emissions of cranberries in response to MeJA application, gypsy moth feeding, and mechanical wounding, and investigate the diurnal pattern of volatile emissions; (3) examine the response of key herbivores (*S. sulfureana* and leafhoppers) and natural enemies [hoverflies (Dip., Syrphidae), minute pirate bugs (Hem., Anthocoridae), spiders (Araneae), and parasitic wasps (Hymenoptera)] to MeJA application on cranberry plants; (4) test the effect of JA-treated cranberry foliage on *S. sulfureana* larval mortality; and, (5) investigate the electrophysiological response of *S. sulfureana* antennae (EAG) to various cranberry leaf volatiles.

## Materials and Methods

### Plants and insects

Cranberries are propagated clonally and grow from single shoots by producing new uprights and runners. Cranberries, *V. macrocarpon* var. Stevens, were grown from rooted cuttings in 10 cm pots in a greenhouse (22 ± 2°C; 70 ± 10% RH; 15:9 L:D) at the Rutgers P. E. Marucci Center for Blueberry and Cranberry Research and Extension (Chatsworth, NJ, USA). Three cuttings were rooted per pot. Plants were allowed to grow in the greenhouse for at least a year before being used in experiments, and were fertilized biweekly with PRO-SOL 20-20-20 N-P-K All Purpose Plant Food (Pro Sol Inc., Ozark, AL, USA) at a rate of 165 ppm N and watered daily. At the time of bioassays, plants from each rooted cutting contained 3–5 uprights and runners. Runners were pruned as needed, and all plants were at the vegetative stage and insect-free when used in experiments.

*Lymantria dispar* caterpillars and *Sparganothis sulfureana* adults and caterpillars were obtained from a laboratory colony maintained at the Rutgers Marucci Center. Caterpillars were reared on a wheat germ diet (Bell et al., 1981) in 30 ml clear plastic cups at 24 ± 1°C, 65% RH, and 14:10 L:D. Field-collected caterpillars were added yearly to the laboratory colony.

### Treatment applications

Plants in each pot were bagged with a spun polyester sleeve (Rockingham Opportunities Corp., Reidsville, NC, USA) and then subjected to one of the following treatments:

(1) Jasmonate treatment: potted plants were sprayed with 1 ml of a 1 mM JA or MeJA solution (Sigma-Aldrich, St. Louis, MO, USA) dissolved in 0.4% acetone or 0.1% Tween 20, respectively. Treated plants were used for bioassays (gene expression, volatile collections, and field experiments, see below) ∼16 h after jasmonate application (unless otherwise stated); this time period was sufficient to induce a volatile response from cranberry leaves in previous studies (Rodriguez-Saona et al., 2011).
(2) Caterpillar treatment: cranberry leaves were damaged by third–fourth instar gypsy moth caterpillars. Four or eight caterpillars were placed on plants and allowed to feed for 2 days before used in bioassays (gene expression and volatile collections); this amount of feeding time by gypsy moth was sufficient to induce a volatile response from cranberry leaves (Rodriguez-Saona et al., 2011).
(3) Mechanical treatment: mechanical damage was inflicted for 2 days by cutting the tips of 40 leaves (20 leaves were mechanically damaged on day 1 and 20 leaves on day 2) with scissors to simulate the amount of leaf area removed by gypsy moth caterpillars. Following, plants were used for gene expression and volatile collections.
(4) Control treatment: plants were either sprayed with 1 ml of distilled water with 0.4% acetone, 0.1% Tween 20, or received no treatment.

Bags were opened just prior to, and closed soon after, treatment.

### Gene expression analysis

Leaves were harvested from jasmonate (MeJA)-treated, caterpillar-damaged, mechanically wounded, and control (Tween) plants (*N* = 3 individual plants or biological replicates per treatment). We used eight rather than four gypsy moth caterpillars in these studies because this herbivore density induces a stronger volatile response in cranberries (see Results). We also harvested undamaged leaves from plants upon which gypsy moths fed and from mechanically wounded plants (systemic response). All samples were collected at 10:00. RNA was immediately extracted from the harvested leaves using the RNeasy Plant Mini Kit (Qiagen; Valencia, CA, USA) according to the manufacturer’s directions. The total RNA was eluted in 100 μl sterile dH_2_O and quantified using the ND-1000 Nanodrop Spectrophotometer (Nanodrop Products; Wilmington, DE, USA). The cDNA was synthesized using 100 ng of RNA per reaction and the Superscript VILO cDNA Synthesis kit (Invitrogen; Carlsbad, CA, USA) according to the manufacturer’s protocol.

The genes targeted for real-time PCR and the primers used for each are listed in Table 1. The eight genes were selected for expression analysis based partly on published reports of terpenoid biosynthesis gene induction when jasmonate treated or in response to herbivores and partly on our own previous work on the volatiles that cranberry plants emit (Rodriguez-Saona et al., 2011). These include (−)-β-caryophyllene synthase (*BCS*), farnesyl diphosphate synthase (*FDS*), *R*-linalool synthase (*LLS*), (*E*)-4-hydroxy-3-methylbut-2-enyl-diphosphate synthase (*MDS*), (3*S*,6*E*)-nerolidol synthase (*NER1*), phosphomevalonate kinase (*PMK*), terpene 1,8-cineole synthase (*TPS*), and α-humulene/β-caryophyllene synthase (*TPS21*). Actin (*ACT*) and RNA Helicase 8 (*RH8*) were used as endogenous controls. The gene sequences were selected from an assembled total genome sequence of cranberry (Georgi et al., 2013 and unpublished). Each target gene sequence was identified by homology to published sequences from other plant species. The primers were designed using the predicted coding sequence of each gene and the Primer Express 3.0 software (Applied Biosystems; Foster City, CA, USA). Real-time PCR reactions were set up using the Power SYBR Green PCR Master Mix (Applied Biosystems) according to manufacturer’s directions and run on an Applied Biosystems 7500 real-time PCR machine. Thermocycling conditions were 50°C–2 min, 95°C–10 min followed by 40 cycles at 95°C–15 s, 60°C–1 min with melt curve set at 95°C–15 s, 60°C–1 min, 95°C–30 s, 60°C–15 s. There were three biological replicates (individual plants) of each sample and three technical replicates were run for each biological replicate. The technical replicates for each biological replicate were averaged. Relative expression levels were calculated by the ΔΔCT method using the DataAssist 3.0 software package (Applied Biosystems) and using *ACT* and *RH8* to normalize the expression.

**Table 1:** **Targets used for real-time qPCR, enzyme commission (EC) numbers and primer sequences**.

Target (abbreviation)	EC number	Real-time F primer	Real-time R primer
Farnesyl diphosphate synthase (FDS)	2.5.1.10	CGAGGTCAGCCATGTTGGTA	CCATCATTTGCAGCAATCAAA
(*E*)-4-hydroxy-3-methylbut-2-enyl-diphosphate synthase (MDS)	1.17.7.1	GCACAGGCGTTACTTCGATTT	CCCTCTTTTTGGATTGGCAAT
Phosphomevalonate kinase (PMK)	2.7.4.2	TTCCCTTCCACCGTTTACATCT	AGGCTTGCGAGTTTCTGAATTT
Terpene 1,8-cineole synthase (TPS)	4.2.3.108	GGTGGGATATAACTGCAATGGAA	TGAAAAGAGCAAGGAAGCAAATC
(−)-β-Caryophyllene synthase (BCS)	4.2.3.57	TCCGGTGGTTACAAAATGCTT	CACCAAATCCCCCATGACA
*R*-Linalool synthase (LLS)	4.2.3.26	GGGCAAGTTTGTGCAATGC	GGCAACTGCCCAGAAGCA
α-Humulene/β-caryophyllene synthase (TPS21)	4.2.3.	TCCGGTGGTTACAAAATGCTT	CACCAAATCCCCCATGACA
(3*S*,6*E*)-nerolidol synthase (NER1)	4.2.3.48	GGGCAAGTTTGTGCAATGC	GGCAACTGCCCAGAAGCA
Actin (ACT)	–	TTCACCACCACGGCTGAAC	AGCCACGTATGCAAGCTTTTC
RNA Helicase-Like 8 (RH8)	3.6.4.13	TGCCAAGATGCTTCAAGATCA	GCATGCACCATTCCGAAAAT

### Headspace collection

Volatiles emitted from cranberry plants that were either damaged by four or eight gypsy moth caterpillars, treated with MeJA, or left undamaged were collected using a push-pull volatile collection system (Tholl and Röse, 2006; Rodriguez-Saona, 2011) in a greenhouse (under conditions described above). The system consisted of four 4 L glass chambers (Analytical Research Systems, Inc., Gainesville, FL, USA). The chambers had a guillotine-like split plate with a hole in the center at the base that closed loosely around the stem of the plant. Incoming purified air entered each chamber at 2 L min^−1^ and was pulled through a filter trap containing 30 mg of Super-Q adsorbent (Alltech; Nicholasville, KY, USA) with a vacuum pump at 1 L min^−1^. Volatile collections were initiated at 10:00. After 4 h of volatile collection, fresh weight of plants was measured to account for differences in plant size. Each treatment was replicated four times.

To determine the diurnal pattern of emissions, volatiles were collected from MeJA-treated at five different times of the day (06:00–10:00, 10:00–14:00, 14:00–18:00, 18:00–22:00, and 22:00–06:00). The experiment was replicated three times.

### Volatile analysis

Collected volatiles were desorbed from the Super-Q traps with dichloromethane (150 μl) and added 400 ng of *n*-octane (Sigma-Aldrich; St. Louis, MO, USA) as internal standard (IS). Headspace samples (1 μl aliquots) were injected onto a Hewlett Packard 6890 Series Gas Chromatograph (GC) equipped with a flame ionization detector and a HP-1 column (10 m × 0.53 mm ID × 2.65 μm film thickness; Agilent Technologies, Palo Alto, CA, USA), with helium flow of 5 ml min^−1^. The temperature program started at 40°C (1 min hold), and rose at 14°C min^−1^ to 180°C (2 min hold), then at 40°C min^−1^ to 200°C (2 min hold). Compounds (relative amounts) were quantified based on comparison of peak areas with that of the IS without FID response factor correction.

Identification of compounds was performed on a Varian 3400 GC coupled to a Finnigan MAT 8230 mass spectrometer (MS) equipped with a MDN-5S column (30 m × 0.32 mm ID × 0.25 μm film thickness; Supelco, Bellefonte, PA, USA). The temperature program was initiated at 35°C (1 min hold), rose at 4°C min^−1^ to 170°C, then at 15°C min^−1^ to 280°C. The MS data were recorded and processed in a Finnigan MAT SS300 data system. The eluted compounds were identified by comparing the mass spectra with those from NIST library spectra and comparison of their retention times to those of commercially available compounds (Rodriguez-Saona et al., 2011).

### Electroantennographic analysis

The relative antennal receptivity of adult males and females of *S. sulfureana* to 11 synthetic volatile compounds emitted from cranberry plants (this study; Rodriguez-Saona et al., 2011) was compared by EAG. The insect head was cut off carefully, and a reference electrode was inserted into its base with a glass capillary filled with physiological saline solution (Malo et al., 2004). The distal end of the antenna was inserted into the tip of the recording glass capillary electrode. EAGs were measured using 5–10 moths of each sex per volatile compound. The signals generated by the antennae were passed through a high-impedance amplifier (NL 1200; Syntech, Hilversum, Netherlands) and displayed on a monitor by Syntech software for processing EAG signals. A stimulus flow controller (CS-05; Syntech) was used to generate a stimulus at 1 min intervals. A current of humidified pure air (0.7 L min^−1^) was constantly directed onto the antenna through a 10-mm-diameter glass tube. Dilutions of the synthetic compounds were prepared in HPLC-grade hexane to make 10 μg per μl solutions. A standard aliquot (1 μl) of each test dilution was pipetted onto a piece of filter paper (0.5 cm × 3.0 cm; Whatman, No. 1), exposed to air for 20 s to allow the solvent to evaporate, then inserted into a glass Pasteur pipette or sample cartridge, and left for 40 s before applying to antennae. A new cartridge was prepared for each insect. To present a stimulus, the pipette tip containing the test compound was inserted through a side hole located at the midpoint of a glass tube through which humidified pure air flowed at 0.5 L min^−1^. The duration of stimulus was 1 s. The continuous flow of clean air through the airflow tube and over the preparation ensured that odors were removed immediately from the vicinity. The synthetic compounds (10 μg) were presented in random order. Control stimuli (air or filter paper with hexane) were presented at the beginning and end of each EAG analysis. Synthetic chemicals were purchased from Sigma-Aldrich and Bedoukian Research (Danbury, CT, USA), and the purities were >95% based on the results with GC.

### Ecological level analysis

The goal of this field study was to investigate the response of arthropods to MeJA-treated cranberry plants. The experiment was conducted in June–July of 2009 to coincide with *S. sulfureana* peak flight activity (Averill and Sylvia, 1998), on a commercial cranberry farm located in Chatsworth, New Jersey (39°72′ N, 74°50′ W), using potted cranberry plants (grown as described above). Potted plants were either treated with MeJA or left untreated (controls) (*N* = 15 pots per treatment). Pots were placed in a 5 × 6 grid pattern within an established cranberry bed (∼2 ha), and were separated by at least 10 m from each other. Treatments were assigned randomly to each location and applied at 17:00. The following morning (08:00), a sticky trap was placed in each pot to monitor abundances of all colonizing arthropods. The sticky traps were 5 cm × 5 cm pieces of green cardboard, covered on both sides (e.g., bi-directional trap) with a thin layer of Tangle-Trap (The Tanglefoot Co., Grand Rapids, MI, USA), and mounted on wooden stakes about 10 cm from the soil, such that the traps were next to but not touching the plants. Traps were left in the field for 48 h, after which they were removed, wrapped in plastic film, and stored in a refrigerator (4°C) for arthropod identification. The entire experiment was replicated three times with a new set of plants each time.

To determine the effects of jasmonate-mediated responses in cranberries on *S. sulfureana* larval survival, an experiment was conducted in a cranberry bed located at the Rutgers Marucci Center. Plots (60 cm × 60 cm) were treated with JA or left untreated (controls), replicated three times, and separated by 60 cm. Applications were made with an R&D (Bellspray Inc., Opelousas, LA, USA) CO_2_ backpack sprayer, using a 1 L plastic bottle, calibrated to deliver 418 L per hectare at 241 kPa (∼15.4 ml per plot). Treatments were applied at 06:00 in early August, 2007. Four hours after treatment, five uprights were randomly collected from each plot and inserted in florists’ water picks, enclosed in a ventilated 40-dram plastic vial, and secured on polystyrene foam trays. Each replicate consisted of a total of 10 vials per treatment (*N* = 30 vials per treatment). Four *S. sulfureana* neonates were added to each vial, and vials were placed in the laboratory at ∼25°C. The number of live larvae was recorded after 7 days.

To confirm that the effects of the JA treatment on *S. sulfureana* larval survival were the result of plant effects rather than any direct toxic effects of JA, an additional experiment was conducted to test the toxicity of JA to *S. sulfureana* neonates. *Sparganothis sulfureana* neonates were placed in 30 ml plastic cups (one neonate per cup), containing 10 ml of wheat germ diet. The diet in each cup was sprayed with either 1 mM JA solution with 0.4% acetone or distilled water with 0.4% acetone 4 h prior to placing the caterpillars (*N* = 50 cups per treatment). The number of live larvae was recorded after 7 days.

### Data analysis

The normalized gene expression levels were log-transformed, to satisfy the homogeneity of variance assumption for ANOVA. However, even with this transformation, responses of two genes (*BCS* and *TPS21*) to one treatment (systemic response in mechanically wounded plants) had excessively large variances, so were excluded from the analyses. Linear mixed models were fit with the lme4 package (Bates et al., 2011) in R (R Development Core Team, 2012) (with biological replicate as a random effect) to a subset of genes whose expression levels changed over treatments, means separations within a gene were done using the multcomp package (Hothorn et al., 2008).

Principal component analysis (PCA) was used to visualize overall differences among blends emitted from each treatment (gypsy moth feeding, MeJA, mechanical wounding, and control) using Minitab v. 16 (Minitab Inc., State College, PA, USA). PCA was initially performed on the data because individual volatile compounds within blends are not independent (Hare, 2011). We also used multivariate analysis of variance (MANOVA; Minitab) to analyze the effects of treatment on volatile emissions. Volatile compounds were grouped into esters, monoterpenes, homoterpenes, sesquiterpenes, or others (only groups containing more than two compounds were considered for MANOVA). A significant MANOVA was followed by ANOVA (Minitab) to determine which compounds within a group were affected by treatment (Scheiner, 2001). Similarly, PCA and ANOVA were used to test the effect of time of day on volatiles emissions from MeJA-treated plants (06:00–10:00, 10:00–14:00, 14:00–18:00, 18:00–22:00, and 22:00–06:00). Volatile emissions data were either ln(*x*)- or ln(*x* + 0.5)-transformed prior to analysis to satisfy assumptions of normality and homogeneity of variances. Differences in the emissions of individual compounds among treatments were analyzed by Tukey *post hoc* comparisons (α = 0.05).

Sticky trap data were first analyzed by functional group, i.e., herbivores and natural enemies (predators and parasitoids), using MANOVA. MANOVA was initially performed on the data because densities of individual arthropod groups are not independent (Scheiner, 2001). The model included treatment (MeJA versus control), time of year (date), and treatment × date. A significant MANOVA was followed by ANOVA for individual arthropod groups. When needed, data were ln(*x*) or ln(*x* + 0.5)-transformed before analysis. Mortality data were arcsine square-root transformed before analysis with ANOVA. A chi-square test was used to determine differences in *S. sulfureana* mortality rate when fed diets sprayed with JA versus unsprayed diets.

The values of the EAG depolarization amplitude after exposure to the volatile compounds were transformed [ln(*x* + 0.5)] prior to analysis with two-way ANOVA with sex of insect and type of chemical compound as the two factors. Significant ANOVA results were followed by Tukey test for means comparison.

## Results

### Gene expression

**Figure 1:**
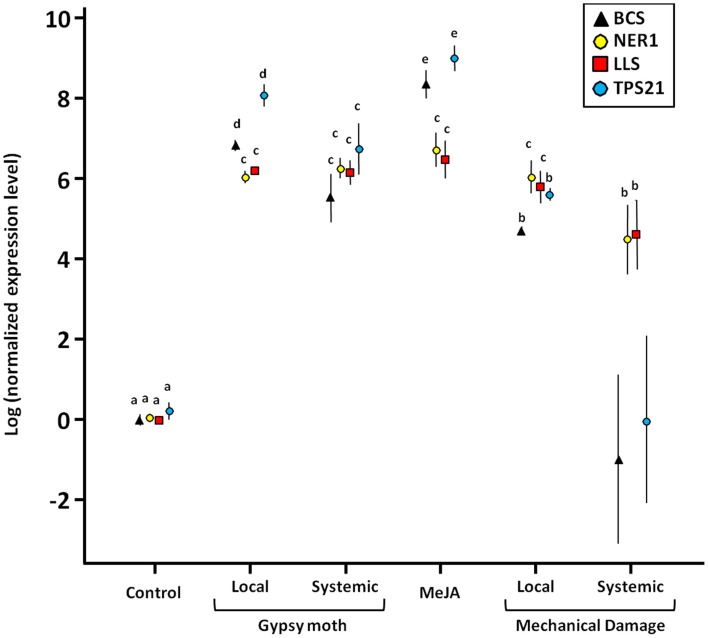
**Expression of terpene genes (*BCS*, *NER1*, *LLS*, and *TPS21*) in cranberries, *Vaccinium macrocarpon*, in response to gypsy moth larval feeding, MeJA, and mechanical damage as compared to control plants**. Targets: *BCS* = (-)-β-caryophyllene synthase; *NER1* = (3*S*,6*E*)-nerolidol synthase; *LLS* = *R*-linalool synthase; *TPS21* = α-humulene/β-caryophyllene synthase. Local = expression at the site of feeding damage or mechanical damage; Systemic = expression of undamaged leaves on damaged plants. Values are the mean ±1 SE. Different letters indicate significant differences among treatments (adjusted for multiple comparisons); there are four sets of means separation letters, one for each gene. *BCS* and *TPS21* values for “Mechanical Damage-Systemic” were not included in the statistical analysis due to excessively high variance.

To determine if the various treatments induced expression of key enzymes in the terpenoid biosynthetic pathway, we tested the relative expression levels of eight selected genes – *BCS*, *FDS*, *LLS*, *MDS*, *NER1*, *PMK*, *TPS*, *TPS21* – using real-time PCR. Four of the genes tested responded significantly to gypsy moth larval feeding, MeJA, and mechanical wounding (*BCS*, *LLS*, *NER1*, and *TPS21*), but to different degrees (Figure 1). The most dramatic changes in expression of *BCS* and *TPS21* were when treated with MeJA. All MeJA-treated plants also had significantly elevated expression of *LLS* and *NER1*. For those genes that responded to gypsy moth feeding (*BCS*, *LLS*, *NER*, and *TPS21*), the changes in gene expression were also evident in the undamaged tissue on the same plant. This was also true in mechanically wounded plants, but only for *NER1* and *LLS*. The undamaged leaves from the mechanically wounded plants had a wide variance in gene expression for *BCS* and *TPS21*. Expression of the other four genes tested (*FDS*, *MDS*, *PMK*, *TPS*) were unchanged for any of the treatments (*P* > 0.05; data not shown).

### Volatile emissions

Cranberry plants responded to gypsy moth feeding in a density-dependent manner such that volatile emissions were ∼2.5 times greater when plants were damaged by eight caterpillars as compared with four caterpillars (four caterpillars [mean emissions in ng h^−1^ g^−1^ fresh tissue ± SE]: 81.9 ± 24.1; eight caterpillars: 199.9 ± 18.4; *F* = 10.41, *df* = 1.6, *P* = 0.018). Eleven out of 22 volatiles were significantly induced by herbivory from cranberry plants compared with undamaged plants (Table 2); with eucalyptol/limonene, linalool, DMNT, indole, β-caryophyllene, α-humulene, and germacrene-D emitted in highest quantities.

**Table 2:** **Volatiles identified in the headspace of cranberry, *Vaccinium macrocarpon*, plants damaged by eight gypsy moth larvae, sprayed with 1 mM MeJA solution, mechanically damaged with scissors, or left undamaged (control)^1^**.

Compound	Control^2^		Gypsy moth		MeJA		Mechanical wounding		*F*^3^	*P*
**LIPOXYGENASE PATHWAY PRODUCTS**										
(Z)-3-Hexenyl acetate^4^	3.8 ± 0.7	(18)b	8.7 ± 1.0	(4)ab	39.2 ± 31.0	(13)ab	313.5 ± 170.6	(92)a	4.88	0.019
**ISOPRENOID PATHWAY PRODUCTS**										
Monoterpenes										
α-Pinene	n.d.	b	n.d.	b	1.5 ± 0.6	(<1)a	n.d.	b	8.10	0.003
Camphene	1.5 ± 0.1	(7)bc	5.1 ± 0.7	(3)a	3.0 ± 0.5	(1)ab	0.7 ± 0.5	(<1)c	10.87	0.001
Sabinene	0.5 ± 0.5	(2)b	4.3 ± 0.8	(2)a	3.3 ± 0.5	(1)ab	9.9 ± 5.2	(3)a	5.56	0.013
β-Pinene	n.d.	b	n.d.	b	1.3 ± 0.4	(<1)a	n.d.	b	8.93	0.002
Myrcene	n.d.	b	2.3 ± 0.3	(1)ab	1.0 ± 0.6	(<1)a	0.3 ± 0.3	(<1) b	7.01	0.006
Eucalyptol/Limonene^5^	0.8 ± 0.4	(4)b	9.2 ± 3.6	(5)a	3.4 ± 0.8	(1)ab	7.3 ± 3.5	(2)ab	5.22	0.015
Linalool oxide	n.d.	b	1.4 ± 0.8	(1)ab	1.5 ± 0.3	(<1)a	n.d.	b	5.93	0.01
Linalool	3.9 ± 0.4	(18)b	23.3 ± 3.6	(12)a	42.6 ± 10.3	(14)a	3.4 ± 0.8	(1)b	35.93	<0.001
Borneol	n.d.	a	n.d.	a	0.4 ± 0.4	(<1)a	n.d.	a	1.00	0.426
Homoterpenes										
α-Copaene	n.d.	b	0.9 ± 0.5	(<1)ab	2.1 ± 0.4	(1)a	n.d.	b	11.66	0.001
β-Cubebene	n.d.	a	n.d.	a	1.6 ± 1.0	(1)a	n.d.	a	2.93	0.077
β-Caryophyllene	n.d.	b	28.0 ± 1.8	(14)a	37.3 ± 18.5	(12)a	n.d.	b	28.71	<0.001
α-Humulene	n.d.	b	15.9 ± 1.1	(8)a	20.6 ± 9.6	(7)a	n.d.	b	41.67	<0.001
Germacrene-D	n.d.	b	8.8 ± 1.0	(4)a	10.6 ± 5.1	(3)a	n.d.	b	32.26	<0.001
α-Farnesene	n.d.	a	n.d.	a	1.1 ± 0.7	(<1)a	n.d.	a	2.89	0.079
δ-Cadinene	n.d.	b	1.4 ± 0.5	(1)a	1.5 ± 0.6	(<1)a	n.d.	b	5.55	0.013
Sesquiterpenes										
4,8-Dimethyl-1,3,7-nonatriene^6^	6.9 ± 1.8	(32)b	57.5 ± 7.4	(29)a	100.0 ± 31.2	(32)a	4.1 ± 0.7	(1)b	45.46	<0.001
**SHIKIMIC ACID /PHENYLPROPANOID PATHWAY PRODUCTS**										
Indole	n.d.	b	15.4 ± 3.0	(8)a	17.2 ± 2.9	(6)a	0.3 ± 0.3	(<1)b	87.98	<0.001
Methyl salicylate	2.8 ± 0.2	(13)ab	3.9 ± 0.2	(2)a	2.7 ± 0.7	(1)ab	1.8 ± 0.4	(1)b	3.88	0.038
Phenylethyl ester	n.d.	b	7.4 ± 0.9	(4)a	6.7 ± 1.5	(2)a	n.d.	b	160.23	<0.001
Benzoic acid, ethyl ester	1.4 ± 0.8	(6)a	6.3 ± 5.2	(3)a	9.4 ± 2.7	(3)a	0.8 ± 0.5	(<1)a	2.66	0.095
Total	21.5 ± 1.3	b	199.9 ± 18.4	a	307.9 ± 71.7	a	342.3 ± 180.6	ab	6.29	0.008

*^1^N = 4*.

*^2^Mean ng n-octane units h^−1^ g^−1^ of fresh tissue (±SE). In parenthesis are percent values based on total amounts. n.d. = not detected (zero values were assigned to non-detectable values for statistical analysis)*.

^3^*df* = 3.12

*^4^For each compound, different letters indicate significant differences between the samples*.

*^5^Peaks of these volatile compounds co-eluted in the GC*.

*^6^This compound was misidentified as myrcenone (based on spectral library match) in Rodriguez-Saona et al. (2011)*.

**Figure 2:**
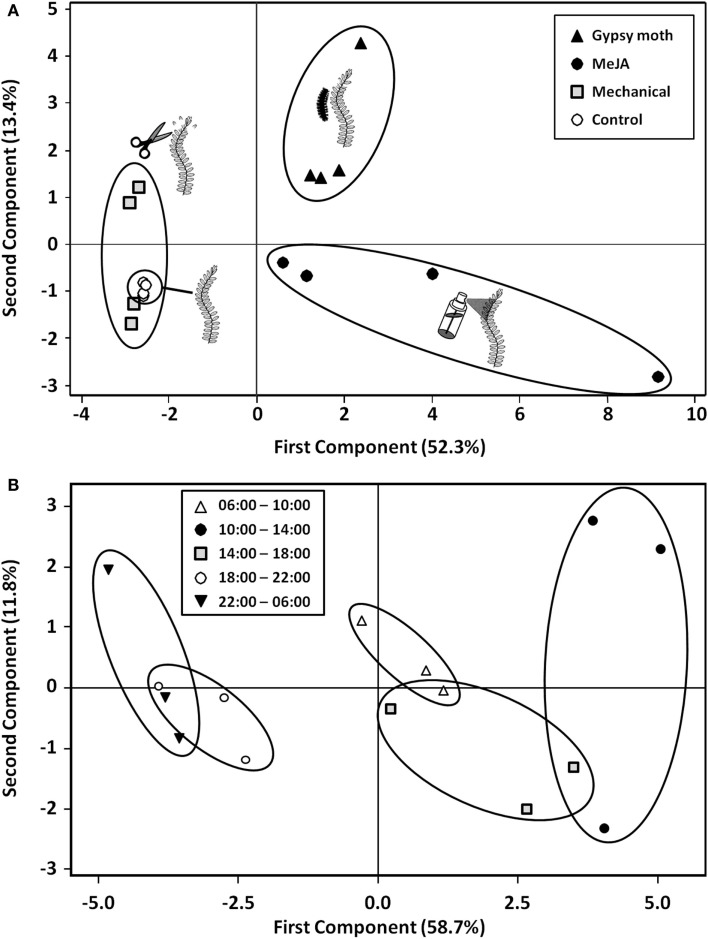
**Score plot of principal component analysis (PCA) for (A) the effects of gypsy moth larval feeding, MeJA, and mechanical damage as compared to control plants on the volatile profiles of cranberries (*Vaccinium macrocarpon*) leaves, and (B) the effects of time of day on volatile emissions from MeJA-treated plants**. Percent variation explained by each principal component is indicated in parenthesis.

Total emissions of volatiles were 9 and 14-fold higher in gypsy moth-damaged (eight caterpillars) and MeJA-treated plants than in control plants, respectively; whereas emissions from mechanically wounded plants were variable and not significantly different from control plants (Table 2). The PCA resulted in a model with the first two PC components explaining 65.7% of the total variation in volatile blends. The score plot of PC1 versus PC2 shows no overlap among volatile blends of gypsy moth, MeJA, and control treatments, while the mechanically wounded and control treatments overlapped (Figure 2A). The first PC component explained 52.3% of the variation in volatile blend and separated the gypsy moth and MeJA treatments from the mechanically wounded and control treatments; while the second PC component explained 13.4% of the variation in the data and separated the gypsy moth from MeJA treatments (Figure 2A).

Two major biosynthetic pathways in the regulation of plant volatiles were influenced by herbivory and MeJA treatments in cranberries; these included the isoprenoid pathway, that resulted in increased emissions of monoterpenes (MANOVA: Wilks’ λ < 0.001; *F* = 4.98; *P* = 0.003), sesquiterpenes (Wilks’ λ < 0.001; *F* = 19.77; *P* < 0.001), and homoterpenes, and the shikimic acid/phenylpropanoid pathway (Wilks’ λ = 0.006; *F* = 11.94; *P* < 0.001). In particular, emission of linalool, DMNT, phenylethyl ester, indole, β-caryophyllene, α-humulene, germacrene-D, and γ-cadinene were higher in the gypsy moth and MeJA treatments compared with the other treatments (Table 2). Only (*Z*)-3-hexenyl acetate emissions were greater in the mechanically wounded plants compared with the control plants (Table 2).

The score plot shows no overlap between volatiles emitted from MeJA-treated cranberry plants at 10:00–14:00 versus those emitted at 06:00–10:00, 18:00–22:00, and 22:00–06:00 (Figure 2B). Total volatile emissions from MeJA-treated cranberries peaked between 10:00 and 14:00 and were lowest between 18:00 and 06:00 (Table 3). Emissions of linalool, DMNT, methyl salicylate, phenylethyl ester, indole, α-copaene, β-cubebene, β-caryophyllene, α-humulene, germacrene-D, and γ-cadinene peaked between 10:00 and 14:00. Only a few compounds (e.g., linalool, DMNT, indole, β-caryophyllene, α-humulene, and germacrene-D) were emitted in detectable amounts at dusk (18:00–22:00) and during the scotophase (in the dark) (Table 3).

**Table 3:** **Time course of volatile emissions from cranberry, *Vaccinium macrocarpon*, plants sprayed with 1 mM MeJA solution^1^**.

Compound	06:00–10:00^2^		10:00–14:00		14:00–18:00		18:00–22:00		22:00–06:00		F^3^	*P*
**LIPOXYGENASE PATHWAY PRODUCTS**												
(Z)-3-Hexenyl acetate^4^	10.3 ± 0.9	ab	13.1 ± 0.7	a	33.9 ± 24.8	a	n.d.	b	n.d.	b	31.41	<0.001
**ISOPRENOID PATHWAY PRODUCTS**												
Monoterpenes										
α-Pinene	n.d.		n.d.		n.d.		n.d.		n.d.		–	–
Camphene	7.8 ± 1.1	a	12.8 ± 2.3	a	11.0 ± 1.3	a	n.d.	b	0.8 ± 0.8	b	28.81	<0.001
Sabinene	2.0 ± 2.2	a	5.2 ± 2.6	a	n.d.	a	n.d.	a	n.d.	a	2.14	0.151
β-Pinene	n.d.	b	4.8 ± 2.4	a	n.d.	b	n.d.	b	n.d.	b	3.98	0.035
Myrcene	n.d.	a	2.7 ± 2.7	a	2.6 ± 2.6	a	n.d.	a	n.d.	a	0.75	0.58
Eucalyptol/Limonene^5^	9.3 ± 2.9	ab	17.0 ± 2.6	a	15.0 ± 2.8	a	1.6 ± 1.6	bc	0.7 ± 0.7	c	9.69	0.002
Linalool oxide	n.d.	a	2.5 ± 2.5	a	n.d.	a	n.d.	a	n.d.	a	1.00	0.452
Linalool	85.7 ± 20.3	a	135.8 ± 23.3	a	52.8 ± 14.4	ab	9.4 ± 0.9	bc	2.5 ± 1.3	c	19.85	<0.001
Borneol	n.d.		n.d.		n.d.		n.d.		n.d.		–	–
Sesquiterpenes										
α-Copaene	5.2 ± 2.8	ab	13.2 ± 1.9	a	10.6 ± 0.7	ab	2.0 ± 2.0	b	0.9 ± 0.9	b	3.75	0.041
β-Cubebene	2.3 ± 2.3	ab	19.0 ± 3.9	a	9.8 ± 4.9	ab	n.d.	b	1.4 ± 1.4	b	3.84	0.038
β-Caryophyllene	123.8 ± 31.0	ab	365.5 ± 104.3	a	264.2 ± 34.3	a	75.1 ± 20.4	ab	43.7 ± 16.6	b	7.63	0.004
α-Humulene	66.4 ± 16.6.6	ab	193.7 ± 55.6	a	152.2 ± 21.5	a	42.5 ± 10.9	ab	25.9 ± 10.2	b	6.68	0.007
Germacrene-D	36.0 ± 10.5.5	ab	104.9 ± 48.1	a	74.1 ± 14.3	ab	18.5 ± 6.7	ab	9.3 ± 4.7	b	3.87	0.038
α-Farnesene	n.d.	a	14.1 ± 7.2	a	8.6 ± 4.3	a	n.d.	a	n.d.	a	3.00	0.072
δ-Cadinene	n.d.	b	17.7 ± 6.8	a	10.0 ± 5.1	a	n.d.	b	n.d.	b	9.30	0.002
Homoterpenes										
4,8-Dimethyl-1,3,7-nonatriene	223.1 ± 75.2	ab	635.8 ± 252.4	a	450.3 ± 128.2	ab	80.2 ± 34.4	bc	24.5 ± 10.5	c	11.90	0.001
**SHIKIMIC ACID/PHENYLPROPANOID PATHWAY PRODUCTS**												
Indole	39.8 ± 4.8	ab	56.8 ± 11.2	a	41.1 ± 12.2	ab	20.0 ± 3.2	b	12.9 ± 2.8	b	8.72	0.003
Methyl salicylate	9.8 ± 1.9	a	11.3 ± 2.8	a	4.5 ± 2.3	ab	n.d.	b	1.2 ± 1.2	ab	6.80	0.007
Phenylethyl ester	13.3 ± 6.8	ab	53.2 ± 18.3	a	15.4 ± 15.4	ab	n.d.	b	n.d.	b	4.49	0.025

*^1^*N* = 3. MeJA was applied 16 h before volatile collections*.

*^2^Mean ng n-octane units h^−1^ g^−1^ of fresh tissue (±SE). n.d. = not detected (zero values were assigned to non-detectable values for statistical analysis)*.

*^3^*df* = 4.10*.

*^4^For each compound, different letters indicate significant differences between the samples*.

*^5^Peaks of these volatile compounds co-eluted in the GC*.

### Electroantennography

**Figure 3:**
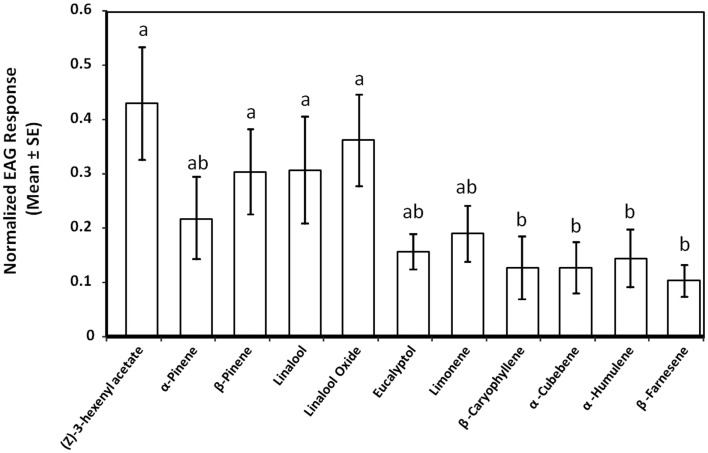
**Electroantennogram (EAG) responses of *Sparganothis* fruitworm, *Sparganothis sulfureana*, to various cranberry, *Vaccinium macrocarpon*, leaf volatiles**. For analysis, control depolarizations (hexane) were subtracted from the test stimuli values. Different letters indicate significant differences among means (*P* ≤ 0.05). Results of male and female *S. sulfureana* responses were combined in the graph (*N* = 13–17).

We used EAG to determine the antennal activity of a polyphagous herbivore, *S. sulfureana*, to various cranberry volatiles. The magnitude of the EAG response was significantly different among the tested chemical compounds (*F* = 2.29, *df* = 10,129, *P* = 0.017). The EAG responses between males and females were, however, not different (*F* = 1.28, *df* = 1,129, *P* = 0.261), nor the interaction between chemical compound and sex of insect (*F* = 0.99, *df* = 10,129, *P* = 0.458). Antennal responses were greater to (*Z*)-3-hexenyl acetate, β-pinene, linalool, and linalool oxide compared with β-caryophyllene, α-cubebene, α-humulene, and β-farnesene (Figure 3).

### Ecological level

Three herbivores were most abundant on sticky traps; these were: the *Sparganothis* fruitworm, *S. sulfureana*, the sharp-nosed leafhopper, *Scaphytopius magdalensis* Provancher (Hem., Cicadellidae), and the blunt-nosed leafhopper, *Limotettix vaccinii* (Van Duzee) (Hem., Cicadellidae). The most abundant groups of natural enemies on traps were hoverflies [mainly *Toxomerus marginatus* (Say)], minute pirate bugs (*Orius* spp.), spiders, and parasitic wasps.

**Figure 4:**
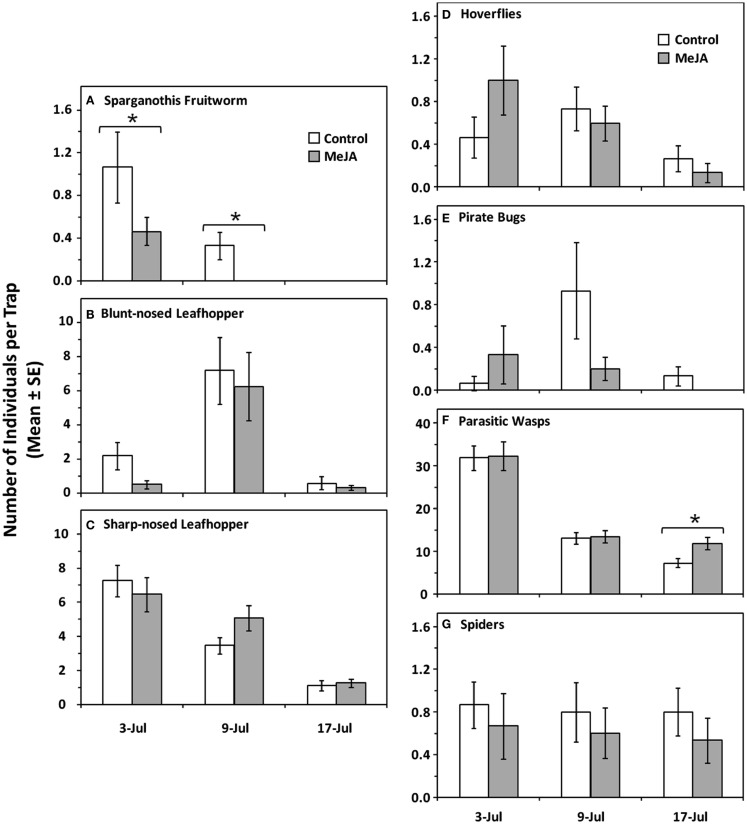
**Mean numbers of arthropods per sticky trap placed near cranberry plants treated with exogenous MeJA and untreated plants (control)**. Herbivores: **(A)** *Sparganothis* fruitworm, *Sparganothis sulfureana* (Lep., Tortricidae), **(B)** blunt-nosed leafhoppers, *Limotettix vaccinii* (Hem., Cicadellidae), **(C)** sharp-nosed leafhopper, *Scaphytopius magdalensis* (Hem., Cicadellidae). Natural enemies: **(D)** hoverflies (mainly *Toxomerus marginatus*) (Dip., Syrphidae), **(E)** minute pirate bugs (*Orius* spp.) (Hem., Anthocoridae), **(F)** parasitic wasps (Hymenoptera), and **(G)** spiders (Araneae). Asterisks (*) indicate statistically significant differences between MeJA and control treatments (*P* ≤ 0.05); all other comparisons were non-significant (*P* > 0.05).

MeJA treatment (MANOVA: Wilks’ λ = 0.86, *F* = 3.36, *df* = 4.81, *P* = 0.014), date (Wilks’ λ = 0.29, *F* = 16.85, *df* = 8.162, *P* < 0.001), but not treatment × date interaction (Wilks’ λ = 0.87, *F* = 1.39, *df* = 8.162, *P* = 0.201) had a significant negative effect on herbivore abundance on sticky traps. When analyzed in more detail, MeJA had an effect on *S. sulfureana* moths (treatment: *F* = 6.10, *df* = 1.84, *P* = 0.016; date: *F* = 13.66, *df* = 2.84, *P* < 0.001; treatment × date interaction: *F* = 1.90, *df* = 2.84; *P* = 0.156) (Figure 4A), but not on the leafhoppers *L. vaccinii* (treatment: *F* = 2.10, *df* = 1.84, *P* = 0.151; date: *F* = 30.83, *df* = 2.84, *P* < 0.001; treatment × date interaction: *F* = 1.06, *df* = 2.84; *P* = 0.352) (Figure 4B), or *S. magdalensis* (treatment: *F* = 1.51, *df* = 1.84, *P* = 0.223; date: *F* = 29.43, *df* = 2.84, *P* < 0.001; treatment × date interaction: *F* = 0.45, *df* = 2.84; *P* = 0.638) (Figure 4C).

Abundance of natural enemies on sticky traps were positively affected by MeJA treatment (MANOVA: Wilks’ λ = 0.68, *F* = 9.122, *df* = 4.81, *P* < 0.001), date (Wilks’ λ = 0.28, *F* = 17.57, *df* = 8.162, *P* < 0.001), and treatment × date interaction (Wilks’ λ = 0.47, *F* = 9.10, *df* = 8.162, *P* < 0.001). When analyzed in more detail, MeJA had a positive effect on parasitic wasps (treatment: *F* = 36.43, *df* = 1.84, *P* < 0.001; date: *F* = 91.56, *df* = 2.84, *P* < 0.001; treatment × date interaction: *F* = 36.48, *df* = 2.84; *P* < 0.001) (Figure 4F), but not on the hoverfly *T. marginatus* (treatment: *F* = 0.31, *df* = 1.84, *P* = 0.582; date: *F* = 4.35, *df* = 2.84, *P* = 0.016; treatment × date interaction: *F* = 1.91, *df* = 2.84; *P* = 0.155) (Figure 4D), minute pirate bugs (treatment: *F* = 1.19, *df* = 1.84, *P* = 0.278; date: *F* = 2.67, *df* = 2.84, *P* = 0.075; treatment × date interaction: *F* = 2.52, *df* = 2.84; *P* = 0.087) (Figure 4E), or spiders (treatment: *F* = 1.21, *df* = 1.84, *P* = 0.275; date: *F* = 0.08, *df* = 2.84, *P* = 0.919; treatment × date interaction: *F* = 0.01, *df* = 2.84; *P* = 0.988) (Figure 4G).

*Sparganothis sulfureana* larval mortality was significantly higher when fed foliage from JA-treated cranberry plants as compared with those fed foliage from untreated plants [mean (± SE) percent mortality = JA: 42.5% (±13.5); control = 0% (±0); *F* = 33.37, *df* = 1.6, *P* = 0.001]. Moreover, the negative effects of JA on larval mortality were attributable to the activation of plant defenses by JA and not to direct toxicity because mortality rates were similar on JA-treated (4%) and untreated diets (8%) (χ^2^ = 0.67; *df* = 1; *P* = 0.414).

## Discussion

Jasmonates such as jasmonic acid and MeJA can serve as an important tool in insect pest management for plant protection against herbivorous pests (Thaler, 1999a; Rohwer and Erwin, 2008; Rodriguez-Saona et al., 2012). Before this can be achieved, however, studies are needed that link the activation of defensive genes by these hormones in plants, the plant’s biochemical changes, and the response of both antagonistic and mutualistic organisms to jasmonate-induced plants. In this study, we showed that: (1) herbivory by gypsy moth caterpillars, jasmonate treatment, and mechanical wounding induce, albeit differently, the expression of genes from two different pathways in the biosynthesis of terpene compounds, i.e., the mevalonic acid (MVA) pathway that produces sesquiterpenes and the methylerythritol phosphate (MEP) pathway that leads to monoterpene production (Lichtenthaler et al., 1997; Paré and Tumlinson, 1999; Bartram et al., 2006; Dudareva et al., 2013); (2) herbivore feeding and jasmonate treatment, but not mechanical wounding, induced emissions of monoterpene, homoterpene, and sesquiterpene volatiles; however, blends were distinct from one another; and, (3) jasmonate treatment reduced preference and performance of the herbivore *S. sulfureana* to cranberry plants, and increased colonization by parasitic wasps. Furthermore, *S. sulfureana* antennae were highly sensitive to inducible monoterpenes, in particular linalool, which might explain the repellence effects of jasmonate-induced plants.

At the molecular level, expression of genes from two isoprenoid biosynthetic pathways – the MVA and MEP pathways – were activated by gypsy moth feeding, MeJA, and mechanical wounding. Of the eight genes targeted in this study – *BCS, FDS, LLS*, *MDS, NER1*, *PMK*, *TPS*, *TPS21*, four responded significantly to gypsy moth larval feeding, MeJA, and mechanical wounding (*BCS*, *LLS*, *NER1*, and *TPS21*), but to different degrees. Expression of the other four genes (*FDS*, *MDS*, *PMK*, and *TPS*) did not change. *MDS*, *PMK*, and *FDS* are higher up in the terpenoid pathway (Terpenoid Backbone Biosynthesis pathway; KEGG map00900), so the whole pathway is not being induced by larval feeding, MeJA, or mechanical wounding but rather the genes involved in the synthesis of specific terpenes. These data thus indicate that cranberry leaves use pre-formed intermediates to rapidly make specific terpenes. The most dramatic changes in expression of *BCS* and *TPS21* were when treated with MeJA; these genes are in the MVA pathway and the enzymes encoded catalyze the production of β-caryophyllene and α-humulene, respectively. Gypsy moth-damaged and MeJA-treated plants also had elevated expression of *LLS* and *NER1* [*LLS* is in the MEP pathway and the encoded enzyme catalyzes the synthesis of linalool; *NER1* is in the homoterpene biosynthesis pathway and the encoded enzyme catalyzes the synthesis of nerolidol-derived DMNT (Boland et al., 1998)]. Interestingly, for those genes that responded to gypsy moth feeding (*BCS*, *LLS*, *NER1*, and *TPS21*), the changes in gene expression were also evident in the undamaged tissue on the same plant, indicating a systemic response in gene expression. This was also true in mechanically wounded plants, but only for *NER1* and *LLS*; thus, genes from the MVA pathway do not appear to be expressed systemically by mechanical wounding.

At the biochemical level, volatiles from the MVA pathway (e.g., β-caryophyllene, α-humulene, and germacrene-D) were strongly induced by gypsy moth feeding but not by mechanical wounding in cranberries. Similarly, volatiles from the MEP pathway (e.g., camphene, eucalyptol/limonene, and linalool) were strongly induced by gypsy moth feeding but not or only weakly induced by mechanical wounding. These data suggest that insect-derived elicitors (see Alborn et al., 1997) are required for the induction of terpene emissions in cranberries. In a previous study, Rodriguez-Saona et al. (2011) found differences in the volatile response to gypsy moth feeding among five cranberry varieties, indicating genotypic variation in the volatile response of cranberries to herbivore feeding. Contrary, they reported similar induction of volatiles by MeJA among these varieties (Rodriguez-Saona et al., 2011). In this study, we also showed that MeJA induces a blend of volatiles in cranberries that was different from herbivore feeding. Other studies have reported a high degree of resemblance, but also some qualitative and quantitative differences, between jasmonate and herbivore-induced volatile profiles (e.g., Dicke et al., 1999; Rodriguez-Saona et al., 2001, 2006; Gols et al., 2003). Altogether, previous data (Rodriguez-Saona et al., 2011) and the present data indicate that signals (i.e., hormones) other than jasmonates might be involved in the emission of HIPVs in cranberries. For example, ethylene was found to interact with JA and the insect-derived elicitor volicitin in the induction of volatile emissions from maize seedlings (Schmelz et al., 2003).

Herbivory and MeJA also induced indole and phenylethyl ester emissions, both products of the shikimic acid pathway (Paré and Tumlinson, 1999), indicating that induced volatiles in cranberries originate mainly from the isoprenoid and shikimic acid pathways. On the other hand, mechanical wounding increased emissions of (*Z*)-3-hexenyl acetate, a product of the lipoxygenase pathway and that is often emitted rapidly in plants as a result of wounding (Paré and Tumlinson, 1999; Chehab et al., 2008).

At the organismal level, jasmonates were involved in the activation of both direct (i.e., plant chemicals that deter or kill the herbivore) and indirect (i.e., chemicals such as HIPVs that attract the herbivores’ natural enemies) resistance in cranberries. Host-plant preference and performance were correlated for the polyphagous herbivore *S. sulfureana*: adults were less attracted to, and larval survival was reduced on, jasmonate-induced plants. Table 4 summarizes studies on the effects of jasmonates on herbivorous arthropod performance and preference – we limit this list to studies that used methodologies similar to ours, i.e., jasmonates (JA or MeJA) were sprayed exogenously on plants; thus, investigated only local responses. Out of 37 herbivore-plant interactions reported in these studies, 31 (84%) showed a negative effect of jasmonates on herbivores, while positive effects accounted for only 8%. Negative effects on the herbivores included lower abundance, performance, colonization, and oviposition on jasmonate-induced plants (Table 4) – likely resulting in increased foraging time and reduced overall fitness; while positive effects included greater abundance, attraction, and oviposition (Table 4). Interestingly, the western flower thrips, *Frankliniella occidentalis* Pergande, performs poorly on tomato (Thaler et al., 2001) and Chinese cabbage (Abe et al., 2009) induced by JA, whereas it has higher performance on JA-induced cotton (Omer et al., 2001). Likewise, the diamondback moth, *Plutella xylostella* L., was more attracted for oviposition to JA-treated compared with untreated common cabbage, while it was less attracted to JA-treated than untreated Chinese cabbage (Lu et al., 2004). Thus, the outcome of these interactions can be host-plant dependent.

**Table 4:** **Effects of jasmonic acid (JA) and methyl jasmonate (MeJA) sprayed exogenously to plants on herbivorous arthropods^1^**.

Arthropod Species	Common Name	Feeding Guild	Elicitor	Concentration (mM)	Ecological Effects	Overall Effect	Plant	Conditions	Reference
*Spodoptera exigua*	Beet armyworm	Chewer	JA	0.5	Lower growth rate/feeding	Negative	Tomato	Laboratory	Thaler et al. (1996)
*Manduca sexta*	Tobacco hornworm	Chewer	JA	1	Lower growth rate	Negative	Tomato	Greenhouse	Cipollini and Redman (1999)
Unknown	Unknown	Chewer	JA	0.5, 1.5	Lower feeding damage	Negative	Tomato	Field	Thaler (1999c)
*Daktulosphaira vitifoliae*	Grape phylloxera	Phloem feeder (roots)	JA	1	Lower performance	Negative	Grape	Greenhouse	Omer et al. (2000)
*Tetranychus pacificus*	Pacific spider mite	Cell-content feeder	JA	1	Lower performance	Negative	Grape	Greenhouse	Omer et al. (2000)
*Spodoptera exigua*	Beet armyworm	Chewer	JA	0.5, 1.5	Lower performance	Negative	Tomato	Field	Thaler et al. (2001)
*Macrosiphum euphorbiae*	Potato aphid	Phloem feeder	JA	0.5, 1.5	Lower abundance	Negative	Tomato	Field	Thaler et al. (2001)
*Myzus persicae*	Green peach aphid	Phloem feeder	JA	0.5, 1.5	Lower abundance	Negative	Tomato	Field	Thaler et al. (2001)
*Frankliniella occidentalis*	Western flower thrips	Cell-content feeder	JA	0.5, 1.5	Lower abundance	Negative	Tomato	Field	Thaler et al. (2001)
*Epitrix hirtipennis*	Flea beetle	Chewer	JA	0.5, 1.5	Lower abundance	Negative	Tomato	Field	Thaler et al. (2001)
*Aphis gossypii*	Cotton aphid	Phloem feeder	JA	1	Lower abundance	Negative	Cotton	Greenhouse/laboratory	Omer et al. (2001)
*Tetranychus urticae*	Two-spotted spider mite	Cell-content feeder	JA	1	Lower abundance	Negative	Cotton	Greenhouse/laboratory	Omer et al. (2001)
*Frankliniella occidentalis*	Western flower thrips	Cell-content feeder	JA	1	Higher abundance	Positive	Cotton	Greenhouse/laboratory	Omer et al. (2001)
*Aphis gossypii*	Aphid nymphs	Phloem feeder	JA	1	Lower performance	Negative	Cotton	Greenhouse/laboratory	Omer et al. (2001)
*Tetranychus urticae*	Two-spotted spider mite	Cell-content feeder	JA	0.1–1	Lower abundance	Negative	Lima bean	Laboratory	Gols et al. (2003)
Unknown	Unknown	Chewer	JA	1	Lower feeding damage	Negative	Lima bean	Field	Heil (2004)
*Plutella xylostella*	Diamonback moth	Nectar feeder	JA	0.01–1	Lower oviposition	Negative	Chinese cabbage	Field/laboratory	Lu et al. (2004)
*Plutella xylostella*	Diamonback moth	Nectar feeder	JA	0.01–1	Higher oviposition	Positive	Common cabbage	Field/laboratory	Lu et al. (2004)
*Macrosiphum euphorbiae*	Potato aphid	Phloem feeder	JA	1.5	Lower performance	Negative	Tomato	Greenhouse	Cooper and Goggin (2005)
*Ips typographus*	Spruce bark beetle	Chewer	MeJA	100	Lower colonization/higher resistance	Negative	Norway spruce	Field	Erbilgin et al. (2006)
*Agrilus planipennis*	Emerald ash borer	Chewer	MeJA	1.4	Higher attraction	Positive	Manchurian ash	Laboratory	Rodriguez-Saona et al. (2006)
*Myzus persicae*	Green peach aphid	Phloem feeder	MeJA	1, 5, 10	Lower population growth	Negative	Tomato	Greenhouse	Boughton et al. (2006)
*Pieris rapae*	Small cabbage white	Nectar feeder	JA	0.1	Lower oviposition	Negative	Brussels sprouts	Greenhouse	Bruinsma et al. (2007)
*Pieris brassicae*	Cabbage white	Nectar feeder	JA	0.1	Lower oviposition	Negative	Brussels sprouts	Greenhouse	Bruinsma et al. (2007)
*Pieris rapae*	Small cabbage white	Nectar feeder	JA	0.5	Lower oviposition	Negative	Black mustard	Greenhouse	Bruinsma et al. (2008)
*Nilaparvata lugens*	Brown planthopper	Phloem feeder	JA	2.5, 5	Increase resistance	Negative	Rice	Greenhouse	Senthil-Nathan et al. (2009)
*Frankliniella occidentalis*	Western flower thrips	Cell-content feeder	JA	0.05	Increase resistance	Negative	Chinese cabbage	Greenhouse	Abe et al. (2009)
*Tetranychus urticae*	Two-spotted spider mite	Cell-content feeder	MeJA	0.1	Increase dispersal/resistance	Negative	Impatiens, Pansy, Tomato	Greenhouse	Rohwer and Erwin (2010)
*Tetranychus urticae*	Two-spotted spider mite	Cell-content feeder	MeJA	N/A^2^	Increase resistance	Negative	Apple, strawberry	Greenhouse	Warabieda and Olszak (2010)
*Dendrolimus superans*	Larch caterpillar moth	Chewer	JA	0.01–1	Lower oviposition	Negative	Larch	Laboratory	Meng et al. (2011)
*Lymantria dispar*	Gypsy moth	Chewer	JA	1	Increase resistance	Negative	Cranberry	Laboratory	Rodriguez-Saona et al. (2011)
*Helicoverpa armigera*	Cotton bollworm	Nectar feeder	MeJA	1.5	No effect on oviposition	None	Tomato	Greenhouse	Tan et al. (2012)
*Gynandrobrotica guerreroensis*	–	Chewer	JA	0.001–1	Repellency (females)	Negative	Lima bean	Laboratory	Ballhorn et al. (2013)
*Cerotoma ruficornis*	–	Chewer	JA	0.001–1	Repellency (females)	Negative	Lima bean	Laboratory	Ballhorn et al. (2013)
*Sparganothis sulfureana*	*Sparganothis* fruitworm	Nectar feeder	MeJA, JA	1	Lower attraction/performance	Negative	Cranberry	Field	This study
*Scaphytopius magdalensis*	Sharp-nosed leafhopper	Phloem feeder	MeJA	1	No effect on abundance	None	Cranberry	Field	This study
*Limotettix vaccinii*	Blunt-nosed leafhopper	Phloem feeder	MeJA	1	No effet on abundance	None	Cranberry	Field	This study

*^1^Listed in chronological order*.

^2^Not indicated

The mechanism of the repellent effects of MeJA on adult *S. sulfureana* remains unknown – it is not known which compound(s) is responsible for these effects. Both male and female *S. sulfureana* antennae responded strongly to four cranberry volatiles [(*Z*)-3-hexenyl acetate, β-pinene, linalool, and linalool oxide]; however, only linalool was emitted in detectable amounts from MeJA-treated cranberry plants during dusk and nighttime (Table 3), when moths are expected to be most active. The more attractive cranberry plants to *S. sulfureana* (controls) emitted low quantities of linalool constitutively; whereas less attractive plants (MeJA-treated) emitted this compound in high amounts (Table 2). This compound has important behavioral effects on other moth species both as an attractant (e.g., Suckling et al., 1996; Raguso et al., 2003) and a repellent (e.g., Kessler and Baldwin, 2001; McCallum et al., 2011), and possibly also on *S. sulfureana*. Also, the herbivore *Spodoptera frugiperda* Smith showed an increment in the antennal response (EAG) to linalool at higher doses (Malo et al., 2004). In our study we could not discard the possibility that jasmonates themselves caused the effect. It was also difficult to determine the gender of the moths on sticky cards because of their poor condition, i.e., moths were either too dry or covered with adhesive. Further studies are needed on the dose-dependent effects of linalool and other induced volatile compounds on *S. sulfureana* foraging behavior, and to determine if gender differences exist in *S. sulfureana*’s response to jasmonate-induced plants and, in particular, the effects of induced volatiles on oviposition.

In contrast to herbivores, the majority (11 out of 18 or 61%) of natural enemy-plant interactions involving jasmonate-induce plants were positive (Table 5). All these interactions involved greater attraction to induced plants likely through increases in volatile emissions. Only 1 out of 18 case studies (6%) reported a negative effect of jasmonates on natural enemies (Table 5). Thaler (2002) reported reduced number of hoverfly eggs laid on induced plants due to a decrease in aphid (prey) abundance. Interestingly, Thaler (2002) found no effect on adult *Hyposoter exiguae* Viereck caught on sticky traps placed beneath the canopy of control and JA-induce tomato plants. However, a previous study (Thaler, 1999b) showed higher parasitism of *Spodoptera exigua* Hübner larvae by *H. exiguae* on JA-induced induced tomato plants, indicating a possible discrepancy between adult attraction and parasitism rate. In the present study, higher numbers of parasitic wasps were caught on sticky traps near MeJA-treated plants than on untreated plants. This attraction coincided with the time of *S. sulfureana* egg laying and larval development; whether this effect translates to greater parasitism of *S. sulfureana* eggs or larvae (or other herbivore pest) requires further investigation. Additional studies are also needed to address the identity and function of these parasitic wasps.

**Table 5:** **Effects of jasmonic acid (JA) and methyl jasmonate (MeJA) sprayed exogenously to plants on natural enemy behavior through induction of volatiles^1^**.

Arthropod species	Common name	Feeding guild	Elicitor	Concentration (mM)	Ecological effects	Overall effect	Plant	Conditions	Reference
*Hyposoter exigua*	Parasitic wasp	Parasitoid	JA	0.5	Higher parasitism	Positive	Tomato	Field	Thaler (1999b)
*Phytoseiulus persimilis*	Predatory mite	Predator	JA	1	Higher attraction	Positive	Lima bean	Laboratory	Dicke et al. (1999)
*Cotesia rubecula*	Parasitic wasp	Parasitoid	JA	1	Higher attraction	Positive	*Arabidopsis thaliana*	Laboratory	van Poecke and Dicke (2002)
Unknown	Hoverfly	Predator	JA	0.5	Lower abundance	Negative	Tomato	Field	Thaler (2002)
*Hyposoter exigua*	Parasitic wasp	Parasitoid	JA	0.5	No effect on adult abundance	None	Tomato	Field	Thaler (2002)
*Hippodamia convergens*	Convergent lady beetle	Predator	JA	0.5	No effect	None	Tomato	Field	Thaler (2002)
*Aphelinid spp*.	Aphid parasitoid	Parasitoid	JA	0.5	No effect	None	Tomato	Field	Thaler (2002)
*Phytoseiulus persimilis*	Predatory mite	Predator	JA	0.1–1	Higher attraction	Positive	Lima bean	Laboratory	Gols et al. (2003)
*Cotesia kariyai*	Parasitic wasp	Parasitoid	JA	1	Higher attraction	Positive	Corn	Laboratory	Ozawa et al. (2004)
*Cotesia glomerata*	Parasitic wasp	Parasitoid	JA	0.5	Higher attraction	Positive	Black mustard	Greenhouse	Bruinsma et al. (2008)
*Eristalis tenax*	Syrphid fly	Predator	JA	0.5	No effect on adults	None	Black mustard	Field	Bruinsma et al. (2008)
*Cotesia glomerata*	Parasitic wasp	Parasitoid	JA	1	Higher attraction	Positive	Brussels sprouts	Laboratory	Bruinsma et al. (2009)
*Cotesia rubecula*	Parasitic wasp	Parasitoid	JA	1	Higher attraction	positive	Brussels sprouts	Laboratory	Bruinsma et al. (2009)
*Diadegma semiclausum*	Parasitic wasp	Parasitoid	JA	1	Higher attraction	Positive	Brussels sprouts	Laboratory	Bruinsma et al. (2009)
*Anastatus japonicas*	Parasitic wasp	Parasitoid	JA	0.01–1	Higher attraction	Positive	Larch	Laboratory	Meng et al. (2011)
unknown (complex)	Parasitic wasp	Parasitoid	MeJA	1	Higher attraction	Positive	Cranberry	Field	This study
*Toxomerus marginatus*	Hoverfly	Predator	MeJA	1	No effect on adults	None	Cranberry	Field	This study
*Orius* spp.	Pirate bug	Predator	MeJA	1	No effect	None	Cranberry	Field	This study

*^1^Listed in chronological order*.

In summary, this study demonstrates the potential of jasmonates as natural plant protectants against herbivorous pests in cranberries. It also summarizes the overall effects of these phytohormones on herbivorous arthropods and their natural enemies (Tables 4 and 5). These studies show that jasmonates provide protection against herbivores from multiple feeding guilds (chewers, phloem feeders, and cell-content feeders) and increase natural enemy attraction in various agro-ecosystems. However, jasmonate-induced responses can be costly in the absence of herbivores (Baldwin, 1998; Cipollini et al., 2003; but see Thaler, 1999c), or could lead to ecological costs due to trade-offs between resistance to herbivores and pathogens (Felton and Korth, 2000). Further studies are needed to measure these costs in cranberries. Other remaining concerns include: the cost of spraying cranberry fields with MeJA, the length of the effect, and the possibility of inducing all plants and producing a no-choice situation.

In addition, this study integrates multiple levels of biological organization from gene expression to biochemical activation to ecological consequences. Such studies are needed for a better understanding of plant-arthropod interactions (Zheng and Dicke, 2008); yet, we are not aware of any similar studies that investigated the effects of jasmonates (e.g., JA or MeJA) on plants and arthropods at all of these three levels of biological organization (molecular, biochemical, and organismal) in a single study (but see Birkett et al., 2000). Our data show general agreement across all three biological levels of organization: key genes from the terpene pathway were highly expressed in MeJA-treated cranberry plants and terpene volatiles were induced by MeJA, which in turn led to repellency of an herbivore and attraction of certain natural enemies. There were, however, some discrepancies: mechanical wounding induced the local expression of four terpene genes – *BCS*, *LLS*, *NER1*, and *TPS21* – that encode the enzymes that catalyze the synthesis of β-caryophyllene, linalool, DMNT, and α-humulene, respectively; however, none of these volatiles were emitted in quantities different from unwounded plants. Therefore, we highlight the need of multiple approaches for a more complete assessment on the effects of various environmental stresses that activate the jasmonate signaling – such as herbivory – on plant-insect interactions.

## Conflict of Interest Statement

The authors declare that the research was conducted in the absence of any commercial or financial relationships that could be construed as a potential conflict of interest.
